# A Groundhog, a Novel *Bartonella* Sequence, and My Father’s Death

**DOI:** 10.3201/eid1512.090206

**Published:** 2009-12

**Authors:** Edward B. Breitschwerdt, Ricardo G. Maggi, Maria Belen Cadenas, Pedro Paulo Vissotto de Paiva Diniz

**Affiliations:** North Carolina State University College of Veterinary Medicine, Raleigh, North Carolina, USA

**Keywords:** Bacteria, Bartonella, dementia, hallucinations, arthritis, vector, antibiotics, infection, disease, another dimension

During the summer of 2007, migratory joint pain developed in my (E.B.B.) 86-year-old father, previously an ironworker, farmer, and World War II veteran. Because of occasional tick attachments, a *Borrelia burgdorferi* ELISA was performed; antibodies were not detected, and no treatment was instituted. In the fall, subtle memory loss developed, and he fell twice a few weeks apart. Dad jokingly blamed the falls and the memory loss on “old timer’s disease.” Subsequently, episodes of subtle confusion and more frequent memory loss generated family concern as to what the future might hold. On December 15, he broke his left femur during a fall while climbing 2 stairs to enter our home. Despite having successfully climbed those stairs thousands of times in the past, he would never climb those or any other stairs again.

Retrospectively obvious, a pattern of insidious illness characterized by joint pain, memory loss, and incoordination, not recognizable by my father or other family members, had begun before that summer. Medically stable historical problems included coronary artery disease, atherosclerosis, carotid artery occlusion, hypertension, and atrial fibrillation. During the previous year, a normocytic, normochromic, nonregenerative anemia persisted. Despite normal serum iron, total iron binding capacity, ferritin, and vitamin B12 values, anemia was attributed to intestinal blood loss. When examined in May 2007, before anesthesia for endoscopy, mood and affect were appropriate, recent and remote memory were intact, insight and judgment were good. A hiatal hernia, mild antral gastritis, and duodenitis were visualized.

## Initial Hospitalization

When my father was hospitalized December 15, 2007, with a broken femur, a resting pill roll tremor and cogwheel rigidity were suggestive of Parkinson disease. Preoperative neurologic consultation identified severe confusion, inattention, and an inability to answer questions. Short-term memory and problem-solving abilities were decreased. There was mild ptosis of the right eye, normal cranial nerves, mild asterixis, and hand weakness. Laboratory abnormalities included anemia, hypercreatinemia, an elevated aspartate aminotransferase level, and hyperglobulinemia. Due to the severity of the femoral fracture, the femoral head was excised and replaced with a bipolar femoral prosthesis.

Postoperatively, poor mentation was considered a sequela of general anesthesia and peri-operative analgesics. For more than a week, dementia persisted. He did not recognize family members and had near constant hallucinogenic activities, including agitation, tying knots, sawing motions, and constantly pulling covers, bed clothes, and fluid lines. Severe hematuria developed after he pulled an inflated Foley catheter from his urethra. Concurrent gastrointestinal bleeding of undetermined cause necessitated multiple blood transfusions. Other complications included difficulty swallowing and paralytic ileus. Repeat abdominal radiographs, in conjunction with stool softeners and laxatives, failed to alleviate gastrointestinal complications. Eventually, he refused food and became severely bloated. Endoscopy performed on December 26 identified severe necrotizing esophagitis, multiple plaques, and a stricture attributed to *Candida albicans* and herpes zoster. *C. albicans* esophagitis is known to accompany HIV infection, leukemia, or an unidentified source of immune suppression (*1*,*2*). Shingles, caused by herpes zoster, occurred during the previous Thanksgiving and can be associated with immunosuppression, stress, or an aging immune system (*3*,*4*). Mentation and gastrointestinal abnormalities improved after starting treatment with fluconazole, acyclovir, and symptomatic medications for erosive esophagitis. However, confusion, lack of orientation, hyponatremia, hypokalemia, and hyperglycemia remained problematic until discharge to a physical therapy center on December 31.

## Second Hospitalization

During the next week, strength and mental capacities improved rapidly and discharge to the home environment was scheduled for January 9. On that morning and while driving to Maryland to build entry ramps, I was informed by cell phone that my father fell out of a chair and became nonverbal and that a stroke was suspected. For me, the roller coaster illness ultimately leading to his death would take an unbelievable turn of events. Upon his transfer to the neurology service, encephalopathy, asterixis, Parkinsonian-type tremor, hypoactive reflexes, pinpoint and minimally reactive pupils, and cogwheel rigidity were found. Verbal communication was absent, but he would grimace with pain whenever extremities were manipulated. An urgent computed tomography scan did not identify intracranial abnormalities.

When I was a boy, my father used the expression “something is fishy in Denmark” to imply that something was astray. Based upon historical events, I suspected something was being missed. Laboratory findings included anemia (hemocrit 34%), a normal leukogram (leukocytes 9,100 cells/μL, 2% band neutrophils), mild hypokalemia, hypoalbuminemia, hyperglycemia, increased serum alkaline phosphatase level, erythrocyte sedimentation rate of 79, and hypergammaglobulinemia. Thoracic radiographs identified mild bilateral pleural effusion. Because an undefined infectious source of immunosuppression seemed plausible, and fever (maximum temperature 38.6°C) occurred 24 hours after neurologic decompensation an infectious etiology was pursued. Nasal methicillin-resistant *Staphylococcus aureus*, blood, urine, and cerebrospinal fluid (CSF) cultures were negative. CSF results, including those for special stains, were unremarkable. Serologic results for *Treponema pallidum, Borrelia burgdorferi*, *Rickettsia rickettsii, Bartonella henselae, Bartonella quintana*, and HIV were negative. Results of CSF herpes simplex PCR and a test for *T. pallidum* antibodies were negative.

On January 11, results of magnetic resonance imaging and magnetic resonance angiography were interpreted as a left posterior stroke with no active bleeding. Initial treatment included intravenous acyclovir, fluconazole, vancomycin, ceftriaxone, ampicillin, and dexamethasone.

## Translational Research and the Practice of Medicine

Because I direct the Intracellular Pathogens Research Laboratory (IPRL) at the North Carolina State University College of Veterinary Medicine, aseptically obtained blood and CSF samples were kindly provided for testing. Results of PCR (*5*,*6*) specific for *Anaplasma, Ehrlichia*, and *Rickettsia* species were negative. *Bartonella* 16S–23S intergenic spacer primers (*7*) repeatedly generated amplicons of different sizes from blood and CSF, respectively. Compared with GenBank sequences, the blood amplicon was most similar (434/465bp) to *Candidatus* Bartonella volans (strain FSq-1, EU294521) isolated from a southern flying squirrel (*Glaucomys volans*) and *Candidatus* Bartonella durdenii (391/422bp) amplified from *Orchopeas howardi* (GenBank accession no. DQ 336386), a flea found on eastern US gray squirrels (*Scinrus carolinensis*), and a *Bartonella* sp. (446/492bp, EF125214) identified in ground squirrels (*Spermophilus danricus*) in the People’s Republic of China. The novel rodent *Bartonella* sequence obtained from my father’s blood had an 18-bp insert at positions 2047 or 334 in EU294521 and DQ336386, respectively. Previously, our laboratory had never worked with rodent *Bartonella* species and had never amplified a 300-bp internal transcribed spacer region amplicon from >3,000 animal or human blood samples. After several unsuccessful cloning attempts, the CSF amplicon was most similar (393/394 bp) to *B. henselae* (NC-005956). Blood and CSF, cultured by using *Bartonella* α Proteobacteria growth medium (BAPGM) (*8*), did not result in the growth of a *Bartonella* species.

## Clinicians and Scientists Working Together

After *Bartonella* PCR results became available, treatment with piperacillin and tazobactam were continued for 3 weeks until discharge. Levetiracetam was added to the patient’s treatment because a generalized seizure occurred shortly after antimicrobial drugs were given. On January 18, severe dependent edema of the right elbow resulted in fluid leakage through intact skin. For 3 weeks, Dad remained semicomatose, disoriented, agitated, and encephalopathic. Hallucinations continued, accompanied by frequent involuntary motor movements. Diabetes mellitus and a large decubital ulcer on the right heel developed. During the fourth week, mentation improved, and he could rise and stand for brief periods. On January 28, he was discharged to a rehabilitation facility, with instructions to receive doxycycline and rifampin 2×/d for 13 days. Blood samples, obtained aseptically before discharge, were again submitted to the IPRL. After BAPGM pre-enrichment and subculture, *B. vinsonii* subsp. *berkhoffii* genotype II was isolated, and sequential serologic testing identified a rising titer to *B. vinsonii* subsp. *berkhoffii* but did not detect *B. henselae* antibodies (Table). After a brief, emotionally traumatic stay at the rehabilitation facility, Dad returned home to be cared for by 4 sons, his wife of 60 years, and other family members. Each week a different son slept by his bed, which was relocated to the family living room.

**Table:** PCR, blood, and CSF *Bartonella* spp. culture and serologic results from an 86-year-old man with recent onset arthritis, memory loss, and encephalopathy*

Date	Location and sample type	Serologic results (titers)			BAPGM enrichment platform		
		*B. henselae*	*B. vinsonii* subsp. *berkhoffii*		Direct extraction	Pre-enrichment culture	*Bartonella* isolate
2005 Sep 23	Home/blood	NA	NA		Neg	Neg	NIO
2006 Aug 19	Home/blood	NA	NA		Neg	Neg	NIO
2008 Jan 11	Hospital 2/blood	NA	NA		*B. volans*–like	Neg	NIO
	Hospital 2/CSF	NT	NT		*B. henselae*	Neg	NIO
2008 Jan 28	Hospital 2/EDTA blood	16	16		Neg	Neg	*Bvb* II
	Hospital 2/ACD blood	NA	NA		Neg	Neg	*Bvb* II
2008 Feb 10	Home/blood	<16	128		Neg	Neg	NIO
2008 Mar 11	Hospital 3/blood	<16	64		Neg	Neg	NIO
2008 Apr 4	Hospital 3/blood	<16	32		*B. volans*–like	Neg	Neg
2008 Apr 28	Home/blood	16	64		Neg	*Bvb* II	Neg

*CSF, cerebrospinal fluid; BAPGM, *Bartonella* α Proteobacteria growth medium; NA, no available serum; Neg, DNA was not amplified by using *Bartonella* 16S–23S intergenic spacer primers; NIO, no isolate obtained by subculture after BAPGM pre-enrichment culture; Hospital 2, second hospitalization; NT, not tested; ACD, acid-citrate-dextrose; *Bvb*II, *B. vinsonii* subsp. *berkhoffii* genotype II; Hospital 3, third hospitalization.

## Home Again at Last

During the next 3 weeks, there was substantial and progressive improvement in physical capabilities and a return of normal mental capabilities, including exceptional short- and long-term memory. Appetite normalized, and despite severe atrophy, muscle strength increased so he could stand, walk with assistance, and, although a daily struggle, access the bathroom. A February 10 blood sample obtained while he was receiving oral antimicrobial drugs was *Bartonella* PCR negative, and no bacteria were isolated in BAPGM. During this precious 3 weeks, our father joked, laughed, and vividly recalled wartime friends and other experiences. On March 1, 2008, he opened Christmas presents with our family. I should have been there.

## Third and Final Hospitalization

On March 4, ≈2 weeks after the course of oral antibiotics was completed, agitation and disorientation returned, and mental status deteriorated. Within 24 hours, Dad was hospitalized, where fine motor tremors of the right hand and wrist, asymmetric edema involving the right leg, and edema of the penis and scrotum were noted. He was afebrile and nonverbal and could not follow simple commands. Hematocrit was 30.2%, platelet count 557,000 cells/μL, and leukocyte count 7,800 cells/μL with a normal differential count. Serum biochemical abnormalities included hyperglycemia (glucose 218 mg/dL), hyperglobulinemia (3.6 g/dL), and increased alkaline phosphatase activity (169 IU/L). Urinalysis abnormalities included proteinuria with occasional hyaline casts.

Lorazepam was administered to control the agitation and restlessness. The warfarin dose was increased and heparinization initiated for a potential cerebrovascular accident. Due to the prior documentation of *Bartonella* infection, intravenous doxycycline, rifampin, and gentamicin were administered, and total parenteral nutrition was instituted. Again, shortly after initiation of antimicrobial drugs, a seizure occurred. *Bartonella* spp. were not amplified or isolated from a 1-mL blood sample obtained 4 days after initiation of treatment with antimicrobial drugs. During the next 3 weeks, while intravenous antimicrobial drugs were administered, our father again remained encephalopathic, with frequent hallucinations, severe agitation, and near-constant mental confusion. On March 14, intravenous methylprednisolone for potential immune-mediated vasculitis elicited no improvement in mental status. Similar to the previous treatment course, improvement in mental status, coherent communication, and renewed ability to recognize family members occurred during the fourth hospitalization week.

## A Battle Lost

On April 4, Dad was discharged to our home and oral antimicrobial drugs (doxycycline and rifampin) were dispensed. Despite all efforts by medical professionals, members of our family, and our tough 86-year-old father, protracted illness and prolonged hospitalizations had resulted in mental and physical debilitation, severe muscle wasting, and profound weakness. More important, he had lost his desire to live. After discharge, there was minimal neurologic improvement. Before the availability of April 4 IPRL test results, he began to refuse all medications. The identical rodent *Bartonella* DNA sequence was again amplified from his blood, but no bacteria were isolated.

Four weeks later my father died, on Friday, May 2, at 5 pm, around quitting time for an old iron worker. My mother and youngest brother were at his side. After his death, blood culture results from another sample obtained by the hospice nurse on April 28, 2008, became available. *B. vinsonii* subsp. *berkhoffii* genotype II was amplified and sequenced from the enrichment BAPGM blood culture. As direct extraction of DNA from blood was negative, growth of viable bacteria in liquid culture was implicated (*8*,*9*).

## Groundhogs, Fleas, and the Genus *Bartonella*

Following our father’s death, I recalled a small, 0.5-cm, raised, firm lesion within his right eyebrow that developed during the summer of 2007 and would spontaneously hurt or burn, causing him to rub or squeeze the lesion. The mass disappeared after he began taking antimicrobial drugs in 2008. Retrospectively, I suspected a rodent flea bite above the eye had transmitted a novel *Bartonella* species, which we sequenced from his blood after each hospitalization. All known *Bartonella* spp. have preferential animal reservoir hosts, and each uses arthropods or animal bites and scratches as the primary modes of transmission (*10*–*12*). Dad would occasionally capture mice, rats, skunks, and groundhogs in the barns. Groundhogs were transported in the car trunk to a distant location for release, potentially leaving behind fleas. Therefore, 3 *Candidatus* Bartonella spp. isolates were provided by Dr. William Nicholson, a colleague at the Centers for Disease Control and Prevention in Atlanta. After sequencing, the 16S–23S intergenic spacer region of a ground squirrel (*Candidatus* Bartonella durdenii), a flying squirrel (*Candidatus* Bartonella volans), and a groundhog (*Candidatus* Bartonella monaxi) isolate, the most similar GenBank sequence was *Candidatus* Bartonella volans. There was no perfect match with these 3 isolates. However, sequences from Dad’s blood clustered with a squirrel *Bartonella* subgroup. This observation supports the presence of a novel *Bartonella* species on the eastern shore of Maryland, an as yet undefined animal reservoir, and an unknown arthropod vector.

Regardless of the mode(s) of transmission, repeated molecular documentation of a novel rodent *Bartonella* sp. and *B. vinsonii* subsp. *berkhoffii* supports the unexpected failure of 2 intensive courses of intravenous and oral antimicrobial drugs to eliminate these fastidious, intravascular bacteria. During the first two hospitalization periods, there was similar and progressive improvement in neurologic signs and mental capabilities that began during the fourth week of antimicrobial drug administration. Pre-enrichment BAPGM growth of *B. vinsonii* subsp. *berkhoffii* from blood obtained 4 days before death supports persistence of viable organisms. Recently, antimicrobial drug resistance genes have been characterized in *B. bacilliformis*, *B. henselae*, and *B. quintana* by in vitro serial passage (*13*–*15*). Retrospectively, the relapse in encephalopathic signs might have been avoided if antimicrobial drugs were continued for a longer interval after discharge from hospital 2, and blood cultures were optimally obtained and sequentially tested to confirm therapeutic elimination.

Elimination of *Bartonella* spp. by antimicrobial drugs in immunocompetent patients may be more difficult to achieve than is currently appreciated (*16*). Although co-infection with *B. henselae* and *B. vinsonii* subsp. *berkhoffii* has been previously reported, DNA of 3 *Bartonella* spp. was detected in our father. Based on repeatable PCR testing, a small quantity of *B. henselae* DNA was in the January CSF sample. Because PCR amplicon contamination was never detected in any negative control, laboratory error is considered unlikely. Although the BAPGM enrichment approach has improved molecular detection and isolation of some *Bartonella* spp. from human patient samples (*9*,*16*–*18*), a rodent *Bartonella* sp. isolate was not obtained. Unfortunately, 8 weeks can be required from inoculation of BAPGM until a subculture agar plate isolate is characterized by DNA sequencing. Therefore, IPRL test results were often not available to Dad’s physicians in a timely manner.

## Age, *Bartonella* spp., and Immune Suppression?

Suspicion of an undetermined source of immune suppression and recent tick exposures were primary factors motivating testing in the IPRL. Previously, *B. vinsonii* subsp. *berkhoffii* was shown to induce immunosuppression in experimentally infected dogs (*19*,*20*). In retrospect, occult infection with *Bartonella* spp. may have contributed to shingles at Thanksgiving and necrotizing *C. albicans* esophagitis after hospitalization for the fractured femur. Recently, *B. quintana* lipopolysaccharide was found to have antiinflammatory properties (*21*). Immune suppressive factors may facilitate persistent intravascular *Bartonella* infection without inducing obvious infection indicators, such as fever, tachycardia, leukocytosis, and CSF pleocytosis. Fever was documented once and mild neutrophilia for 3 of 48 blood counts. Thrombocytosis, previously associated with *B. henselae* (*22*), was documented 14 times.

## Ecologic Complexity of *Bartonella* spp.

Because of my father’s long-standing atherosclerosis and because BAPGM will grow a spectrum of seemingly difficult to isolate bacteria (*8*,*23*), pre-enrichment blood cultures and *Bartonella* internal transcribed spacer region PCR had been performed in September 2005 and August 2006 (Table). *Bartonella* spp. were not amplified or isolated, which suggests infection occurred after the summer of 2006. Transmission of *B. henselae, B. vinsonii* subsp. *berkhoffii*, and *B. alsatica* can occur as a result of a scratch from a cat, a dog, or a wild rabbit, respectively (*17*,*24*–*26*). Cats are the primary reservoir for *B. henselae*, whereas dogs and coyotes are the only reported reservoir hosts for *B. vinsonii* subsp. *berkhoffii* genotype II in North America (*27*). Recently, *B. vinsonii* subsp. *berkhoffii* genotype II was isolated by BAPGM blood culture from a cat with recurrent osteomyelitis (E.B. Breitschwerdt, unpub. data), which suggests that a bacteremic cat might facilitate transmission of this subspecies. My parents had an old (≈21 years of age) exclusively outdoor barn cat that would occasionally scratch. The cat could not be tested because it died in 2007. *B. henselae* and *B. clarridgeae* have been transmitted experimentally by transfusion to cats (*24*). Because *B. vinsonii* subsp. *berkhoffii* seroconversion occurred during hospitalization, transfusion-associated transmission is also possible. The exact timing and mode of transmission of *B. henselae, B. vinsonii* subsp. *berkhoffii*, and the rodent *Bartonella* sp. to our father cannot be established. However, his illness serves to illustrate the medical and ecologic complexity of this genus.

## Occult Infection and Chronic Illness

Reconstructing the history of a chronic illness is always difficult and remains an unexacting science due to known, unknown, and undetermined factors that influence disease expression over time. Experimental studies that used rodent models have emphasized the ability of *Bartonella* spp. to invade erythrocytes and vascular endothelial cells (*28*). In vitro *studies* indicate that *B. henselae* can infect macrophages, microgial cells, dendritic cells, and CD34+ progenitor cells (*29*). *B. henselae* and *B. vinsonii* subsp. *berkhoffii* have been amplified from dog lymph node aspiration samples (*30*). Thus in a given patient, *Bartonella* organisms likely infect a substantial number of cellular targets. *B. henselae* infection induces chronic arthritis in a subset of cat scratch disease (CSD) patients, and atypical CSD manifestations are more likely to develop in elderly patients (*31*,*32*). In the context of arthritis, *B. henselae* and *B. vinsonii* subsp. *berkhoffii* were repeatedly isolated from joint fluid from a dog in which repeated antimicrobial drug therapy was not successful (*33*). Although a spectrum of acute and generally self-limiting neurologic manifestations have been historically described in CSD patients, *B. henselae* and *B. vinsonii* subsp. *berkhoffii* were only recently isolated from patients with chronic neurologic and neurocognitive abnormalities (*16*).

We propose that the initial arthritic signs, short-term memory loss, and incoordination were premonitory signs of *Bartonella* spp. infection, and that persistent infection contributed to localized edema, nonregenerative anemia, thrombocytosis, hyperglobulinemia, and a protracted debilitating illness accompanied by hallucinations, agitation, seizures, and death. Agitation, disorientation, and combative behavior have been reported in association with CSD and physicians have implicated *Bartonella* spp. as contributors to agitation and treatment-resistant depression (*34*,*35*). Memory loss and a spectrum of neurocognitive complaints have also been reported in immunocompetent persons infected with *B. vinsonii* subsp. *berkhoffii* and *B. henselae* (*9*,*16*,*17*).

## My Father and “One Medicine”

In recent years, there has been renewed interest in the concept of “One Medicine” (*36*). I hope that lessons from my father’s death can reinforce the importance of “One Medicine.” However, as in the past, rhetoric may not result in needed increases in resource allocation to enhance educational, research, service, and public health capabilities of the veterinary profession (*37*). In the context of vector-borne infectious diseases, zoonotic diseases, food safety, zoologic medicine, and environmental medicine and ecosystem health, to name a few areas, veterinarians continue to make major contributions that ensure and enhance the daily health of animals and humans. Because of a research focus in comparative infectious diseases and knowledge of the biologic, immunologic, and pathophysiologic behavior of *Bartonella* spp. in a spectrum of animal species, a veterinary research laboratory was able to assist with the management of my father’s illness.

As is often true of research at the bedside and the laboratory bench, new lessons and challenges arose from the collective efforts of doctors, nurses, veterinarians, research scientists, and others attempting to heal my father. I and my family remain sincerely grateful to the many doctors, nurses, and other caregivers who contributed to the management of my father’s surgical and medical problems. Because of the severe encephalopathic and combative nature of his behavior, this was frequently not an easy or pleasant task. During his illness, I was struck by several items: 1) most nurses are absolutely amazing, caring, and dedicated professionals; 2) in human medicine, unlike veterinary medicine, no physician claimed or accepted the responsibility to be my father’s doctor; and 3) for many reasons, I found the human healthcare system to be frayed, if not broken. Whether blame lies with the insurance companies, our litigious society, the profit-based motivations of hospital administration, the increased complexity of medical technology, or the medical education of physicians, it really does not matter. As my father would say, “It is no way to do business.” I have taught internal medicine at a College of Veterinary Medicine for 32 years. During that time, every sick animal on our medical service had at least 2 doctors (1 being a student), who were directly responsible for the animal’s care, for frequent communications with the owner, and for communications with the referring veterinarian. In our increasingly complex hospital environments, every patient needs a personal advocate or designated doctor to represent his or her interests.

## Epilogue

Some years ago in a conversation with my mother I suggested that the term natural death may well represent an oversimplification of the processes that end a person’s life. My father and our family were substantially affected during his illness. Each day, from initial hospitalization until his death, there was at least 1 family member at his side. In the eyes of family and friends, my father was a great man in so many respects. He was a loving husband, a caring father, diligent worker, and a friend and supporter to many persons. Potentially, his illness illustrates a complex interaction between intravascular *Bartonella* infection and complex disease expression, provides documentation for an as yet uncharacterized zoonotic rodent *Bartonella* sp., and offers disconcerting evidence supporting antimicrobial drug ineffectiveness and clinical evidence supporting the concept that persistent infection with >1 *Bartonella* spp. may lead to immunosuppression and opportunistic infections with organisms such as herpes zoster and *C. albicans*. As in his life, Dad would want this story to benefit others after his death. We hope that it does.
